# Plant membranes and border control

**DOI:** 10.1093/jxb/erx229

**Published:** 2017-08-09

**Authors:** Angus Murphy

**Affiliations:** University of Maryland, College Park, MD, USA

**Keywords:** CHUKNORRIS, conserved function, endomembrane system, membrane, neofunctionalization, TAA/YUC biosynthesis, transporter, vacuole

**This special issue brings together current topics in plant membrane biology, from the vacuole as a homeostatic storage body for nutrients and small molecules, through characterization of membrane transporters (including an exploration of neofunctionalization and conserved function), to the endomembrane localization of proteins. Among many highlights are the localization of components of the auxin TAA/YUC biosynthetic pathway and a new system, CHUKNORRIS, (ironically) a genuine example of the sort of tool essential for moving into large-scale interpretation of the analog data that characterizes so many aspects of biological function.**

It is highly likely that encapsulation of an ordered set of macromolecules within a self-assembling enclosure composed of amphipathic hydrocarbons was an early and recurrent event in the emergence of living systems on this planet. These boundaries provided the opportunity to impose order on the inherent entropy of an aqueous population of macromolecules and to create an environment supporting chemiosmotic potentials to drive energetic exchanges. As in all other organisms, molecular exchanges and signaling events at endomembranes and the plasmalemma are essential to plant growth and acclimation to local environments. Our understanding of this interplay has developed rapidly in recent years, facilitated by researchers sharing ideas and working collaboratively (Box 1).

**Box 1. A half century of membrane biology**

For more than fifty years, the International Workshop on Plant Membrane Biology (IWPMB) has served as an independent forum for discussion of plant membrane research, including the roles that membrane proteins play in physiology and development. Consider the progress over this period: the fluid mosaic model was only proposed 45 years ago (Singer and Nicolson, 1972).

Although originally a forum for analyses of ion movement, characterizations of transporter proteins, mineral nutrition, and salt stress studies, it now also covers topics such as membrane protein trafficking, structural biology, transcriptomic analyses of membrane function, and breakthrough technology used to study membrane function. In 2016 the 17th IWPMB was held in Annapolis, Maryland (USA), and research presented and discussed inspired this special issue of *Journal of Experimental Botany* (*JXB*). Recent related special issues of the journal include ‘Membrane–Protein Interactions in Plants: Why, When and How?’ and ‘Plant Nuclear Envelope, Nuclear Structure and Nucleoskeleton’ (see Graumann, 2015; also academic.oup.com/jxb for all the special issues).

It has been more than 25 years since our understanding of the plant vacuole as a multifunctional organelle rather than ‘cellular garbage dump’ began to emerge. Research has now shown that the vacuolar proton ATPase regulates cellular elongation and trafficking of membrane proteins through the endomembrane system, and the vacuole itself functions as a homeostatic storage body for nutrients and small molecules (Martinoia *et al.*, 2012; Schumacher, 2014). The mechanisms that sort protein cargo to the plasma membrane or vacuole via the prevacuolar compartment have been exquisitely characterized, and those molecular dissections have revolutionized our understanding of plant cell biology. More recently, auxin repression of actin-dependent vacuolar expansion has been shown to inhibit cell elongation (Scheuring *et al.*, 2016). In this special issue, Yang *et al.* (2017) summarize current understanding of the vacuole as a regulator of phosphorus homeostasis and highlight the contributions that recently characterized orthophosphate transporters make to vacuolar polyphosphate and phytate sequestration.

The vacuole is also important in plant responses to salt stress, and understanding these responses has become increasingly important as soil salinity associated with non-sustainable agricultural practices and limiting water resources has become more prevalent. Chloride has been viewed as both an essential nutrient and toxicant for some time (White and Broadley, 2001), and in this issue Wege *et al.* (2017) review the recent literature to highlight the sometimes-forgotten role of chloride in plant physiology (see also Franco-Navarro *et al.*, 2016; Raven, 2017). Furthermore, Qiu *et al.* (2017) describe the characterization of the SLAC anion channel homolog SLAH1 and its role in modulating salt tolerance and chloride accumulation in the shoot. Last, Han *et al.* (2017) review the phenomenon of seedling halotropism and describe what is known about this salt-avoidance mechanism and whether salt regulation of the PIN2 auxin efflux carrier is regulated exclusively by phosphorylation and lipid signaling or also involves interactions with chloride.

## Family ties and infidelities

The enormous amount of information generated by genomics and transcriptomics has led to the creation of large catalogs of membrane transporters and regulatory proteins. However, transporter function is often extrapolated from biochemical assessments conducted with founding members of a protein family, and subsequent physiological and developmental analyses of the functions of some transporters suggest that family members may not share functionality. Corratgé-Faillie and Lacombe (2017) review emerging evidence that many NTR1/PTR FAMILY (NPF) proteins transport substrates other than nitrate and oligopeptides. As described by the authors, NPFs have now been shown to transport the plant hormones auxin, abscisic acid, jasmonate, and gibberellins, as well as glucosinolates. Multiple roles in biotic interactions have also been described. Xu *et al.* (2017) show that a small family of GTR transporters is required for movement of glucosinolates and their catabolites from sites of production in the stele to the root cortex.

Another theme is neofunctionalization within transporter families as ‘secondary’ transporter characteristics are selectively promoted to primary status. For instance, the ATP-Binding Cassette (ABC) superfamily is associated with both general xenobiotic detoxification and more selective transport, and structural studies have helped elucidate the basis of substrate specificity (Bailly *et al.*, 2011). All ABC subclass B and C (ABCB/ABCC) transporters, including mammalian PGP/ABCB1 and the Cystic Fibrosis Transmembrane Receptor, exhibit greater or lesser multi-substrate transport activity dependent on pore size as well as channel-like activity for small anions or cations (Gill *et al.*, 1992; Luckie *et al.*, 2003; Chen and Hwang, 2008; Yang and Murphy, 2009; Cho *et al.*, 2014). In contrast, plant and fungal ABCG transporters share a discrete origin, and function primarily in transport of hydrophobic substrates including hormones, waxes, and xenobiotics (Box 2). In this issue, Biała *et al.* (2017) demonstrate that SBCG10 from *Medicago truncatula* transports phenylpropanoid precursors in the medicarpin biosynthetic pathway.

**Box 2. New ABCG structural information supports preference for a broad range of hydrophobic substrates**

Although ABCB and ABCG transporters share a common structure comprising two nucleotide-binding/hydrolysis and two transmembrane domains, plant ABCBs exhibit a more limited preference for simple and aromatic organic acids compared to ABCGs. The recent solution of multiple crystal B and G class ABC proteins allows for structural comparison that highlights the obvious differences underlying functional divergence of the respective subgroups. The presence of members of both subgroups across all eukaryotic phyla supports ancient divergence of the two ABC subclasses.

Left: Arabidopsis auxin transporter ABCB19 threaded on *Thermotoga maritima* TM287-288 (PDB: 4Q4A). Middle: Arabidopsis ABCG36 threaded on the human sterol transporter ABCG5/ABCG8 heterodimer (PDB: 5DO7). Right: Arabidopsis cuticular lipid transporter ABCG11 homodimer threaded on the human sterol transporter ABCG5/ABCG8 heterodimer (PDB: 5DO7). The gray box represents the approximate position of the plasma membrane. All models built using Modeller v9.14. The author would like to thank Mark Jenness for providing the computational analysis shown.

**Figure:**
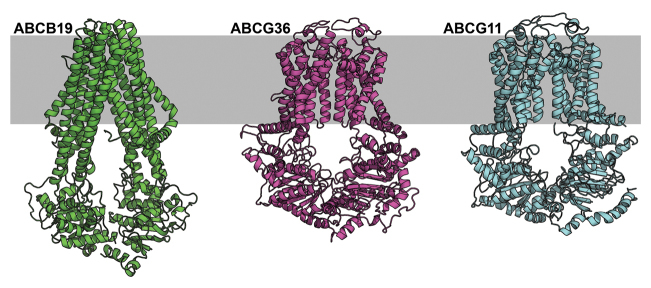

It is also important to consider the conserved functions of transporters. The research paper by Padmanabhan *et al.* (2017) describes developmental control of pollen and vegetative growth by conserved modulation of pH and potassium homeostasis by CHX transporters. Wigoda *et al.* (2017) provide intriguing evidence that differential gene expression in the bundle sheath compared to mesophyll tissue underlies differential AKT2-like membrane potassium conductances. Similarly, Wang *et al.* (2017) demonstrate that conserved functioning of GORK potassium channels coupled with Respiratory Burst Oxidase Homolog D (RBOHD) activity is vital to hypoxia acclimation in Arabidopsis. Costa *et al.* (2017) demonstrate that phosphorylation of the plasma membrane calcium ATPase transporter ACA8 does directly modulate the calcium signature *in planta*. Finally, a contribution from Luan *et al.* (2017) reviews the groupings of potassium and phosphate transporters and regulatory proteins, and the practical need to regulate these networks of proteins if crop production is to be sustainable.

The role of the complex chloroplast membrane system in regulating exchanges of organic and inorganic ions in photosynthesis is well characterized. However, the interactions of photosynthetic and photoprotective mechanisms with ion transport within the chloroplast are still poorly understood. Szabò and Spetea (2017) summarize current understanding of chloroplast ion transport, and Bose *et al.* (2017) describe the modifications of ion transport observed in halophytes and propose how discrete transporters protect chloroplasts under extreme conditions.

## Location, location, location

One of the most exciting reports in this issue is the localization of components of the auxin TAA/YUC biosynthetic pathway to the endoplasmic reticulum (ER) and endomembrane system. Kriechbaumer *et al.* (2017) use the tools of cell biology to demonstrate that Tryptophan Amino Transferase and the YUCCA indole-3-acetate synthase co-localize together in the ER. Although YUCCA6 was previously suggested to have an endomembrane localization (Kim *et al.*, 2007), most depictions of auxin biosynthetic pathways place the enzymes in the cytosol, perhaps because earlier reports had associated auxin oxidative catabolism with microsomal membranes. However, Zhang and Peer (2017) present a review highlighting new evidence that physiologically relevant auxin oxidase activity is cytosolic. Another spatial concern is resolved by the localization of the dolichol kinase DOK1 to the ER by Cho *et al.* (2017). The presumed dependence of dolichol signaling on glycosylation is consistent with ER localization and high levels of expression in meristematic and young tissues. Organization of proteins within membrane structures also involves ordered membrane domains and protein modifications, especially at the plasma membrane. Hemsley (2017) reviews our current understanding of the role of S-acylation in membrane complex stabilization. Finally, a paper by Sabol *et al.* (2017) describes the regulation of the exocyst subunit Exo70B by RIN4, providing a rationale for the function of RIN4 in localizing pathogen defense responses.

## Looking ahead

Making sense of complex data is the generational task of our time as researchers. Damineli *et al.* (2017) describe a new system, called CHUKNORRIS, to interpret oscillatory signatures and make transformations to quantitative results that can be compared with growth parameters (see also Simon Gilroy’s accompanying Insight article discussing the importance of this methodology: Gilroy, 2017). Users can easily ignore the reactionary name and employ the system to rapidly interpret oscillatory measurements, eliminate or disqualify noisy data, and enhance experimental efficiency. Such tools are essential if we are to move beyond the relatively simple digital datasets that comprise genomics and transcriptomics and move into large-scale interpretation of the analog data that characterizes so many aspects of biological function.
